# Host genetics impact on SARS-CoV-2 vaccine-induced immunoglobulin levels and dynamics: The role of *TP53*, *ABO*, *APOE*, *ACE2*, *HLA-A*, and *CRP* genes

**DOI:** 10.3389/fgene.2022.1028081

**Published:** 2022-11-30

**Authors:** Donato Gemmati, Giovanna Longo, Ines Gallo, Juliana Araujo Silva, Paola Secchiero, Giorgio Zauli, Stefania Hanau, Angelina Passaro, Patrizia Pellegatti, Stefano Pizzicotti, Maria Luisa Serino, Ajay Vikram Singh, Veronica Tisato

**Affiliations:** ^1^ Department of Translational Medicine, University of Ferrara, Ferrara, Italy; ^2^ Centre Haemostasis & Thrombosis, University of Ferrara, Ferrara, Italy; ^3^ University Centre for Gender Medicine Studies, University of Ferrara, Ferrara, Italy; ^4^ Research Department, King Khaled Eye Specialist Hospital, Riyadh, Saudi Arabia; ^5^ Department of Neuroscience & Rehabilitation, University of Ferrara, Ferrara, Italy; ^6^ Hospital-University of Ferrara, Ferrara, Italy; ^7^ Department of Chemical & Product Safety, German Federal Institute for Risk Assessment, Berlin, Germany

**Keywords:** SARS-CoV-2, COVID-19, SNPs, vaccine, BNT162b2 (Pfizer–BioNTech), ChAdOx1 (AstraZeneca), vaccine pharmacogenomics, COVIDomics

## Abstract

**Background:** Development and worldwide availability of safe and effective vaccines against severe acute respiratory syndrome coronavirus-2 (SARS-CoV-2) to fight severe symptoms of coronavirus disease 2019 (COVID-19) and block the pandemic have been a great achievement and stimulated researchers on understanding the efficacy and duration of different vaccine types.

**Methods:** We investigated the levels of anti-SARS-CoV-2 antibodies (IgG) and neutralizing antibodies (NAbs) in 195 healthy adult subjects belonging to the staff of the University-Hospital of Ferrara (Italy) starting from 15 days up to 190 days (about 6 months) after the second dose of the BNT162b2 (Pfizer–BioNTech) mRNA-based vaccine (n = 128) or ChAdOx1 (AstraZeneca) adenovirus-based vaccine (n = 67) using a combined approach of serological and genomics investigations.

**Results:** A strong correlation between IgG and NAb levels was detected during the 190 days of follow-up (*r* ^*2*^ = 0.807; *p* < 0.0001) and was confirmed during the first 90 days (T1) after vaccination (*r* ^*2*^ = 0.789; *p* = 0.0001) and 91–190 days (T2) after vaccination (*r* ^*2*^ = 0.764; *p* = 0.0001) for both vaccine types (*r* ^*2*^ = 0.842; *p* = 0.0001 and *r* ^*2*^ = 0.780; *p* = 0.0001 for mRNA- and adenovirus-based vaccine, respectively). In addition to age (*p* < 0.01), sex (*p* = 0.03), and type of vaccine (*p* < 0.0001), which partially accounted for the remarkable individual differences observed in the antibody levels and dynamics, interesting genetic determinants appeared as significant modifiers of both IgG and NAb responses among the selected genes investigated (*TP53*, rs1042522; *APOE*, rs7412/rs429358; *ABO*, rs657152; *ACE2*, rs2285666; *HLA-A* rs2571381/rs2499; *CRP*, rs2808635/rs876538; *LZTFL1*, rs35044562; *OAS3*, rs10735079; *SLC6A20*, rs11385942; *CFH*, rs1061170; and *ACE1*, ins/del, rs4646994). In detail, regression analysis and mean antibody level comparison yielded appreciable differences after genotype stratification (P_1_ and P_2_, respectively, for IgG and NAb distribution) in the whole cohort and/or in the mRNA-based vaccine in the following genes: *TP53*, rs1042522 (P_1_ = 0.03; P_2_ = 0.04); *ABO*, rs657152 (P_1_ = 0.01; P_2_ = 0.03); *APOE*, rs7412/rs429358 (P_1_ = 0.0018; P_2_ = 0.0002); *ACE2*, rs2285666 (P_1_ = 0.014; P_2_ = 0.009); *HLA-A*, rs2571381/rs2499 (P_1_ = 0.02; P_2_ = 0.03); and *CRP*, rs2808635/rs876538 (P_1_ = 0.01 and P_2_ = 0.09).

**Conclusion:** High- or low-responsive subjects can be identified among healthy adult vaccinated subjects after targeted genetic screening. This suggests that favorable genetic backgrounds may support the progression of an effective vaccine-induced immune response, though no definite conclusions can be drawn on the real effectiveness ascribed to a specific vaccine or to the different extent of a genotype-driven humoral response. The interplay between data from the polygenic predictive markers and serological screening stratified by demogeographic information can help to recognize the individual humoral response, accounting for ethnic and geographical differences, in both COVID-19 and anti-SARS-CoV-2 vaccinations.

## Introduction

In November 2019, the first cases of coronavirus disease 2019 (COVID-19) due to severe acute respiratory syndrome coronavirus-2 (SARS-CoV-2) infection were diagnosed in Wuhan (China). The virus sequence was released in January 2020 after 54 days from the first declared case, and in March 2020, 63 days after the SARS-CoV-2 sequence was published, the first vaccines were already in the trial phase (Tregoning et al., 2020). In September 2020, the vaccine landscape included 43 candidate formulations under clinical trial evaluation, and the European Medicines Agency (EMA) released authorizations for Pfizer/BioNTech, Moderna, AstraZeneca, and Janssen Pharmaceutical. Two different main strategies of vaccine formulation have been adopted, adenoviral vector-based vaccine (e.g., AstraZeneca or Janssen Pharmaceutical) and mRNA-based vaccine (e.g., Pfizer–BioNTech or Moderna), potentially offering similar efficacy and safety (Falsey et al., 2021; Thomas et al., 2021).

At the beginning of 2021, vaccines seemed to be the complete remedy, even though the presence of conventional mild side effects such as local reactions at the injection site, fever, and fatigue. In addition, some rare episode of vaccine-associated thromboses or more severe vaccine-induced immune thrombocytopenia and thrombosis (VITT) with intracranial hemorrhage and death have been reported in few individuals after adenoviral vector-based vaccine (Polack et al., 2020; Baden et al., 2021; Pavord et al., 2021; Elberry et al., 2022; Klok et al., 2022).

After the first months of the vaccination campaign, novel concerns and questions addressed the efficacy and duration of the circulating antibodies against SARS-CoV-2 induced by vaccines, and this complex issue was the source of alarm among clinicians, researchers, and population, considering the upcoming booster dose scheduled before the end of 2021. Interindividual variables, such as host genetics, sex, age, and different vaccine formulations and the appearance of different spike variants, could be responsible for the observed different antibody response to vaccines. Interestingly, specific *HLA* regions have been previously recognized by genome-wide association studies (GWAS) as genetic effectors of the interindividual variability in immunoglobulin levels and in response categorization to hepatitis-B vaccine, ascribing to few selected loci, which play a role in the regulation of the humoral immunity (Jonsson et al., 2017; Chung et al., 2019). The association between the *HLA* system and COVID-19 prognosis has been thoroughly investigated, recognizing potential *HLA* carriers associated to higher risk of death (Hamming et al., 2004; Grifoni et al., 2020; Huang et al., 2020; Liao et al., 2020; Pretti et al., 2020; Tay et al., 2020; Wang et al., 2020; Zhang et al., 2020). Although recent studies suggested that *HLA* polymorphisms might unlikely account for the wide disparities in COVID-19 prognosis, they concluded on the potential role of *HLA* polymorphisms on the development of SARS-CoV-2 immunity also after vaccination by evaluating the association between specific *HLA* variants and vaccine response (Copley et al., 2021; Shukla et al., 2022). Even though a first extensive report excluded the *HLA* impact on the immunoglobulin level fluctuation and duration (Ragone et al., 2021), particular attention has been later directed toward a selected locus (HLA-A*03:01) associated to a weak antibody response and negative side effects after Pfizer–BioNTech vaccine (Bolze et al., 2022; Crocchiolo et al., 2022).

Although the connection between the *ABO* locus and SARS-CoV-2 susceptibility has been clearly established (COVID-19 Host Genetics Initiative, 2021; COVID-19 Host Genetics Initiative, 2022), additional studies investigating the possible effect of the ABO blood type on the risk of SARS-CoV-2 infection, COVID-19 prognosis, and antibody response to anti-SARS-CoV-2 vaccine or infection yielded conflicting results (Gil-Manso et al., 2022; Sgherza et al., 2022; Vicentini et al., 2022; Ziberna et al., 2022). Blood group O has been reported as a possible protective factor for disease progression (Pereira et al., 2022), and recent studies have ascribed to the ABO locus a modifier role on the antibody titers, showing lower levels to B and O types compared to A and AB (de Freitas Dutra et al., 2021). Conversely, higher neutralizing antibody levels have been found to characterize B blood group subjects when compared to the remaining blood groups (Bloch et al., 2021). Moreover, though intriguing hypotheses and mechanisms have been proposed, specific research works addressing the relationships between the phenotypic ABO blood group and anti-SARS-CoV-2 vaccine response did not reach definite results (Sgherza et al., 2022). Finally, positive associations between C-reactive protein (CRP) and immune response after SARS-CoV-2 infection or vaccine have been recently reported, hypothesizing basal or unresolved inflammation as causative reasons (Salvagno et al., 2022a; Gianfagna et al., 2022).

Host genetics and acquired factors modulate the clinical phenotypes of COVID-19 by complex interactions occurring between SARS-CoV-2 and human cells and represent issues investigated by the COVID-19 Host Genetics Initiative (HGI) (Severe Covid et al., 2020; COVID-19 Host Genetics Initiative, 2021); moreover, host genetics may also give precious information on the strength of the individual vaccine immune response and efficacy (Jonsson et al., 2017; Colucci et al., 2021; Castro Dopico and Karlsson Hedestam, 2022; Venkataraman et al., 2022). It is to be noted that the same panel of inherited and acquired factors or combinations of them seems to contribute to both COVID-19 progression and vaccine immune response by sharing common mechanisms and pathways (Gemmati et al., 2020; Gemmati and Tisato, 2020; Tiwari Pandey et al., 2020; Zauli et al., 2020; Cuapio et al., 2022; Milani et al., 2022).

To select in advance individuals at risk with no proper SARS-CoV-2 immune response, it would be extremely useful to predict the development of a weak/strong or a short/long-lasting anti-SARS-CoV-2 immunity either by natural infection or vaccination. In the present study, we assessed the levels of anti-SARS-CoV-2 IgG and neutralizing antibodies in a cohort of adult healthy vaccinated subjects and stratified the results by selected variants in key genes and locus, previously investigated in the COVID-19 prognosis, or in vaccine-induced humoral and cell-mediated response, or as causative of specific mild/common adverse reactions among vaccinated subjects. In detail, the following genes and locus have been considered: *ACE*, *ACE2*, *ABO*, *APOE*, *CFH*, *CRP*, *HLA-A*, *LZTFL1*, *OAS3*, *SLC6A20*, and *TP53*, to disclose the puzzled architecture of the interindividual antibody response and dynamics to anti-SARS-CoV-2 vaccine.

## Materials and methods

### Study design

A retrospective study aimed at assessing the immune response in healthy adult volunteers (n = 195) belonging to the staff of the University-Hospital of Ferrara vaccinated with two doses of anti-SARS-CoV-2 vaccine: Pfizer–BioNTech/BNT162b2 (mRNA-based vaccine) or adenovector, ChAdOx1/AstraZeneca (AdV-based vaccine), in the period starting from January 2021, according to the directives of the Italian Health Ministry. A 6-month period from the second vaccine dose (15–190 days) was considered adequate to monitor the entire circulating antibody kinetic as recently reported by Salvagno et al. in healthcare workers (Salvagno et al., 2022b). The study involving human participants was reviewed and approved by the local regional ethical committee (CE-AVEC; 405/2020/Oss/UniFe); the participants provided their written informed consent to participate in this study. The study consisted of a first immunological assessment aimed at evaluating the anti-SARS-CoV-2 circulating antibodies (IgG and neutralizing antibodies, NAbs) and a genotyping profile investigation including a group of selected common gene variants to identify candidate genetic modifiers of the vaccine-induced immune response.

### Blood sample and DNA extraction

Whole blood samples were collected in specific vacutainers, and plasma samples were processed within 1 h from drawing blood, and they were immediately frozen at -80 °C in multiple aliquots and blind tested. DNA was isolated from frozen whole blood by using an automated DNA extraction and purification robot (BioRobot EZ1 system, Qiagen; Hilden, Germany).

### Antibody assay

Anti-SARS-CoV-2 immunoglobulin-G (IgG) levels were assessed in duplicate by using Human SARS-CoV-2 Spike (Trimer) IgG-ELISA kit (Invitrogen, Thermo Fisher Scientific, United States) and neutralizing antibodies (NAbs) by SARS-CoV-2 neutralizing antibody ELISA kit (Invitrogen, Thermo Fisher Scientific, United States) in the previously frozen plasma samples, following the manufacturers’ instructions by using a Tecan Infinite MPlex reader (Tecan Trading AG, Switzerland), as previously described (Agostinis et al., 2012).

### PCR and pyrosequencing

PCR detection of the selected gene variants was as follows: *ACE2* (rs2285666; G>A), *HLA-A* (rs2571381; T>C, and rs2499; T>G), *LZTFL1* (rs35044562; A>G), *OAS3* (rs10735079; G>A), and *TP53* (P72R, rs1042522; G>C), by rhAmp SNP genotyping technology (IDT, Integrated DNA Technologies, Coralville, IA, United States) on the QuantStudio3 Real-Time PCR System (Thermo Fisher Scientific, United States), according to the supplier’s instructions; *ABO* (rs657152; A>C), *APOE* (R158C, rs7412; C>T, and C112R, rs429358; T>C), *CFH* (Y402H, rs1061170; C>T), *CRP* (rs2808635; G>T; rs876538; T>C), and *SLC6A20* (rs11385942; -/A), by pyrosequencing (Pyromark ID System, Biotage, AB, Uppsala, Sweden) after standard PCR on Agilent SureCycler 8800 (Agilent Technologies, Santa Clara, CA, United States); and *ACE1* (ins/del, rs4646994), by agarose gel electrophoresis (I/D allele: 490bp/190bp). DNA samples with known genotypes were used as internal control references for all the sequencing, and a random number of samples (15% for each genotype) were reanalyzed as the internal quality control procedure as previously described (Gemmati et al., 2004; Zamboni and Gemmati, 2007).

### Statistical analysis

Statistical analyses were performed using GraphPad Prism version 8 (GraphPad Software, Inc.) and MedCalc version 20.113 (MedCalc Software Ltd.). Regression analyses and scatter diagrams accounted for antibody levels and genetic stratification analysis, respectively; *p*-values for slope and regression equations accounted for the comparison differences. Moreover, Welch’s *t*-test for antibody levels comparison, Chi-square test for genotype distributions or allele frequency, and Hardy–Weinberg equilibrium to check possible deviation of genotype/allele distribution were also used according to the recent study of Colucci et al. (2021). The Mann–Whitney non-parametric paired test was performed in case of multiple blood samples from the same donors over time, to evaluate the changes in antibody levels. Spearman’s test was used to assess correlation analyses. *p*-values were two-sided with a threshold for statistical significance fixed to *p* ≤ 0.05.

## Results

### Study population

We recruited 195 healthy subjects among the worker staff at the University-Hospital of Ferrara starting from January 2021 at least 15 days after the second vaccine dose (15–190 days) and before the booster dose of anti-SARS-CoV-2 vaccine. Table 1 shows the epidemiological characteristics of the vaccinated subjects stratified by the two main vaccine formulations received: mRNA-based vaccine, n = 128 (Pfizer–BioNTech) and AdV-based vaccine n = 67 (AstraZeneca). Moreover, a subgroup of 52 subjects (Pfizer–BioNTech, n = 32 and AstraZeneca, n = 20) recruited among those who had the blood sampling within the first 3 months from the second dose of vaccine, kindly agreed to undergo a second blood drawn scheduled about 90 days from their first sampling and they were separately computed for coupled analyses.

**TABLE 1:** Baseline characteristics of the subjects involved in the study.

		Whole cohort (n = 195)		*P*
		mRNA-based vaccine	Vector-based vaccine	
n (%)		128 (65.6)	67 (34.4)	
**Age (years)** **^a^**		53.5 (44–60.5)	50 (42–59)	ns
**Females**	**n (%)**	70 (54.7)	32 (47.7)	ns
	**Age (years)** **^a^**	53.5 (47.5–59.5)	51.5 (44.5–58.5)	
**Males**	**n (%)**	58 (45.3)	35 (52.3)	ns
	**Age (years)** **^a^**	54 (45.5–60.5)	54 (46.5–60.5)	
***P*** (females vs*.* males)		ns	ns	

^a^ Median (IQ, range); ns indicates not significant.

### Antibody levels (IgG and NAbs)

**FIGURE 1:**
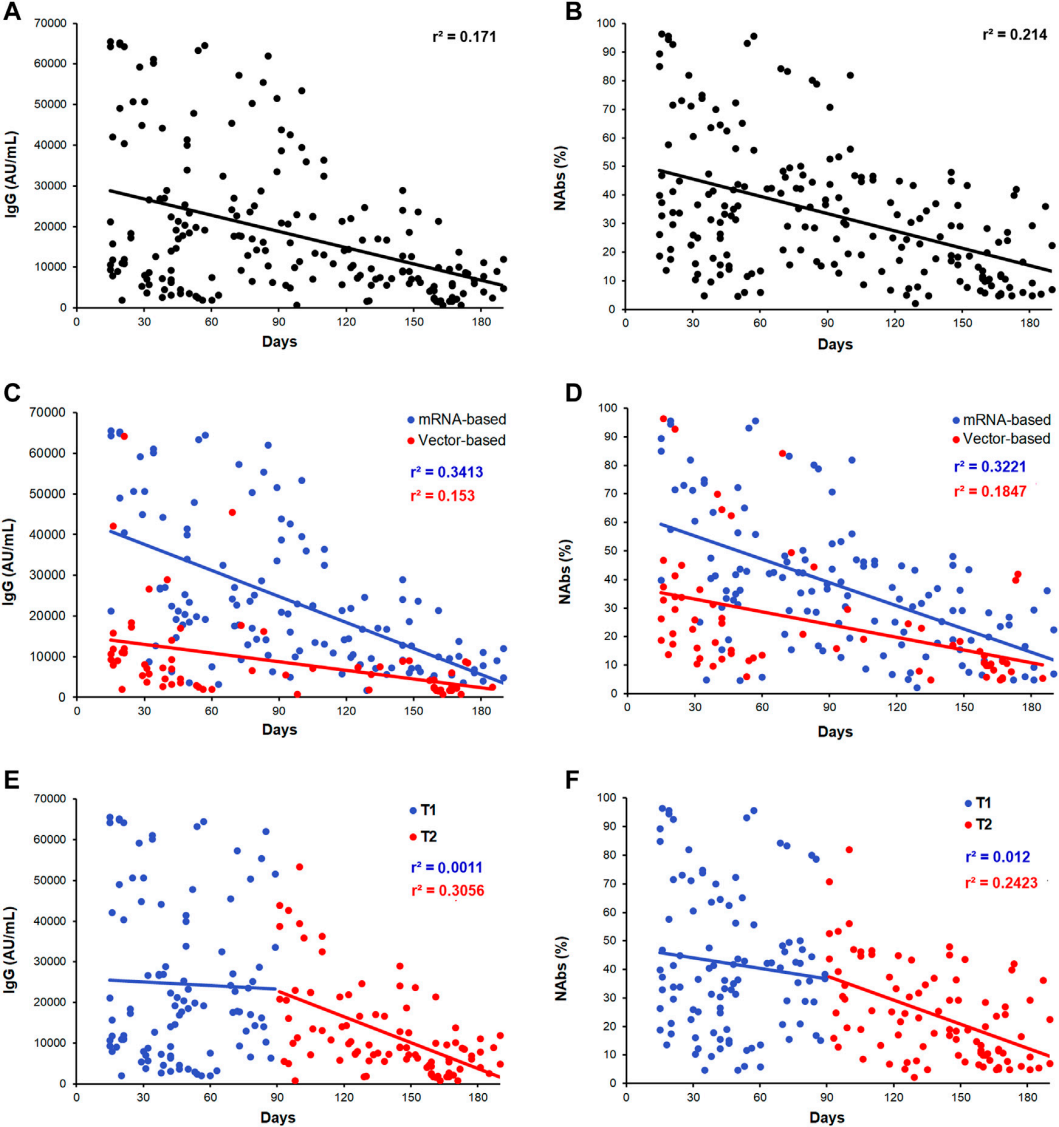
Scatter plots of the distribution of antibody levels after second dose of vaccine. **(A,B)** IgG and NAbs kinetic distributions in the whole cohort of subjects. **(C,D)** IgG and NAbs distribution stratified by the vaccine type (mRNA-based vaccine: blue dots/line and vector-based vaccine: red dots/line). **(E,F)** IgG and NAbs distribution, respectively, stratified by T1 and T2 recruitment time (T1: blue dots/line and T2: red dots/line). Each panel shows the specific regression line and the *r*-coefficient.

Figure 1 shows the levels distribution and dynamic of anti-SARS-CoV-2 IgG (Figure 1A) and NAbs (Figure 1B) in the whole cohort of vaccinated subjects investigated over a period of 15–190 days after the second dose of vaccine. We next split subjects into those whose blood was collected within 90 days (T1; n = 104) and after 90 days (T2; n = 91) from the second dose of anti-SARS-CoV-2 vaccine. As expected, a significant decrease of antibody titer has been observed during the time considered. Regardless the time frame between the day of vaccination and the day of blood sampling, IgG and NAbs showed a wide range in levels, suggesting a strong interindividual variability. Globally, a significant fall in levels within the T1–T2 period was observed (IgG, *r* ^*2*^ = 0.171; *p* < 0.0001 and NAbs, *r* ^*2*^ = 0.214; *p* < 0.0001). By comparing the two vaccine types, mRNA-based vaccine vs. AdV-based vaccine, different distributions for both IgG and NAbs were evident (Figures 1C,D). The T1–T2 median time of blood sampling among the mRNA- and AdV-vaccinated subjects was 91.0 days (min 15 days and max 190 days) and 89.5 days (min 15 days and max 185 days), respectively. For a more appropriate evaluation, the two vaccine formulations were also separately assessed for T1 and T2 time points. Accordingly, the specific kinetics and dynamic distributions for IgG levels were characterized by *r* ^2^ = 0.341, *p* < 0.001 and *r* ^2^ = 0.153, *p* < 0.001, while for NAbs levels by *r* ^2^ = 0.322, *p* < 0.0001 and *r* ^2^ = 0.184, *p* < 0.0001, respectively, for the mRNA-based vaccine and AdV-based vaccine (Figures 1C,D).

By considering a sex stratification, the same analysis showed slightly higher antibody distributions in females than in males for both vaccine formulations (IgG, *p* = 0.03 and NAbs, *p* = 0.06 in the whole cohort; IgG, *p* = 0.10 and NAbs, *p* = 0.24 in the mRNA vaccine subgroup, and IgG, *p* = 0.42 and NAbs, *p* = 0.58 in the AdV vaccine subgroup).

Moreover, as shown in Figures 1E,F, a significant fall in antibody levels was more evident after 3 months from vaccination, being negligible during the first 90 days (T1 blue dots) compared to that observed after 90 days (T2, red dots): IgG: T1, *r* ^2^ = 0.001; *p* = 0.74 and T2, *r* ^2^ = 0.305; *p* < 0.0001; NAbs: T1, *r* ^2^ = 0.012; *p* = 0.09 and T2, *r* ^2^ = 0.242; *p* = 0.001).

**FIGURE 2:**
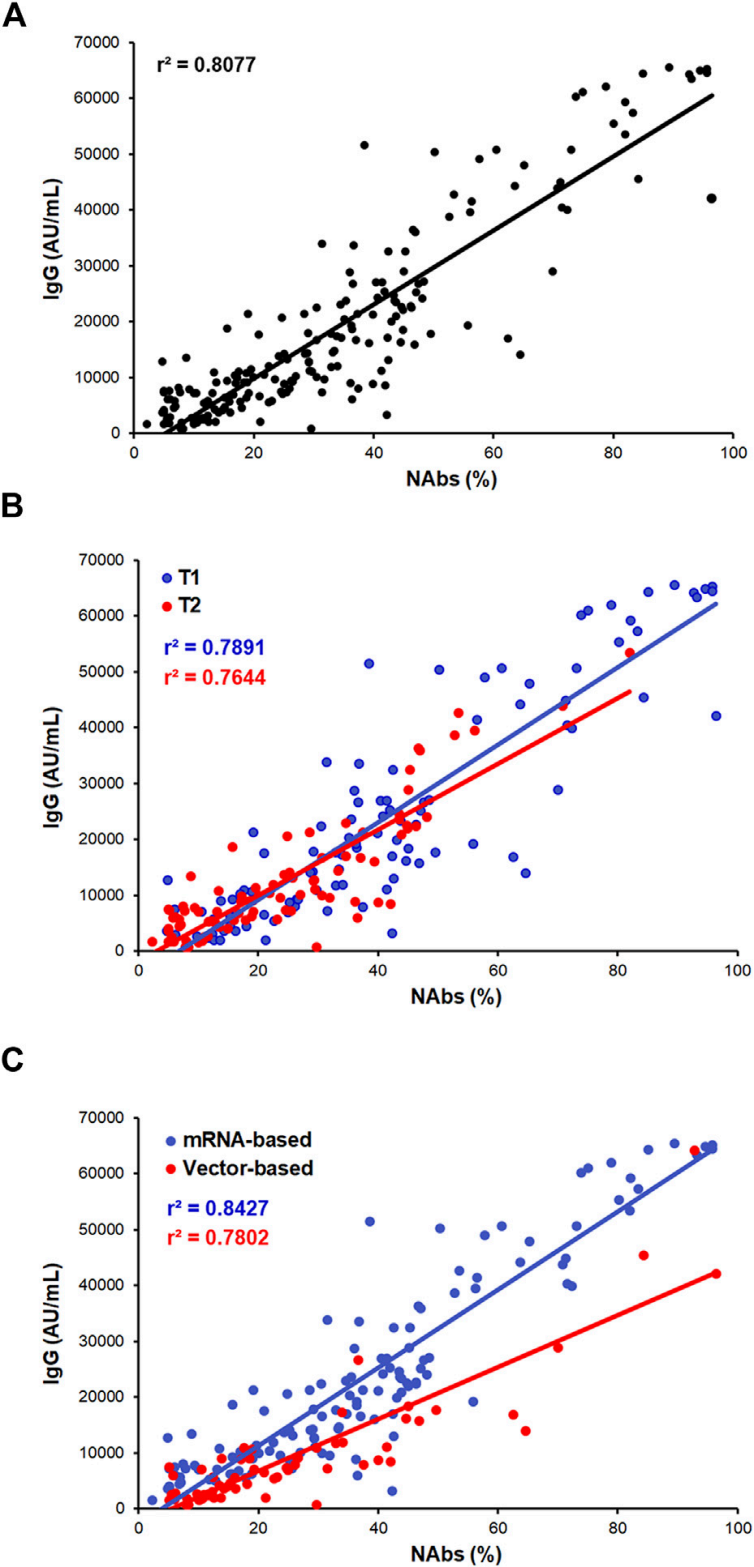
IgG and NAbs correlation analysis. **(A)** Correlation between IgG and NAbs level distributions in the whole cohort of subjects. **(B)** Correlation between IgG and NAbs stratified by T1 and T2 (T1: blue dots/line and T2: red dots/line). **(C)** Correlation between IgG and NAbs stratified by vaccine formulation (mRNA-based vaccine: blue dots/line and vector-based vaccine: red dots/line). Each panel shows the specific regression line and *r*-coefficient.

A strong correlation between IgG and NAbs levels was found in the whole cohort of samples during the whole frame of time considered (Figure 2A; *r* ^2^ = 0.807; *p* < 0.0001) and within the T1 and T2 time points (Figure 2B; *r* ^2^ = 0.789; *p* = 0.0001 and *r* ^2^ = 0.764; *p* = 0.0001, respectively). Finally, even though the strong difference in the antibodies level distribution detected between the two vaccine types, both vaccines maintained a strong IgG/NAbs correlation when separately assessed (Figure 2C; *r* ^2^ = 0.842; *p* = 0.0001 and *r* ^2^ = 0.780; *p* = 0.0001 for mRNA- and AdV-based vaccine, respectively).

The wide variability observed in the antibodies distribution, especially due to the different vaccine type, sex, and the timing of blood sampling, prompted the researchers to perform a detailed assessment of the antibody levels by a pairwise analysis of IgG and NAbs in 52 vaccinated subjects (32 mRNA-based vaccine, ♀46.5%, and 20 AdV-based vaccine, ♀45%), who agreed to have double sampling. The T1–T2 median time of the blood sampling in the 52 subjects was comparable to that of the 195 subjects of the whole cohort in order to lessen the time-dependent effects on the antibody’s level (89.5 days, min 15 days and max 187 days, and 88.0 days, min 15 days and max 185 days) for mRNA- and AdV-vaccinated subjects, respectively. Supplementary Figures S1A-D show the comparison of IgG and NAbs levels analyzed for T1 and T2 in the two vaccine formulations, respectively, for the whole cohort of 195 subjects and the subgroup of 52 blood-paired sampling. The sub-analysis in the 52-paired subjects confirmed what is being observed in the whole cohort, with no significant differences in the levels of antibodies comparing the two cohorts matched by the vaccine type.

### Gene variants and antibody distribution

We further explored the impact of common genetic variants within genes previously investigated as promising modifiers of the clinical phenotype and progression of COVID-19 or as possible influencers of the antibody response after SARS-CoV-2 infection or vaccination. Some of them have also been investigated in the variability of humoral and cell-mediated response or as causative of specific mild/common adverse reactions, particularly among Pfizer/BNT162b2 vaccinated subjects. Table 2 shows in detail the list of the selected gene variants investigated in our cohort of subjects.

**TABLE 2:** Panel of gene and variants investigated.

Gene symbol	Gene ID	aa or nt change	SNP (rs)
*ABO*	28	A>C,T	657152
*ACE1*	1636	del/ins	1799752
*ACE2*	59272	C>A,G,T	2285666
*APOE*	348	C>T	7412
		T>C	429358
*CFH*	3075	C>A,G,T	1061170
*CRP*	1401	G>C,T	2808635
		T>A,C,G	876538
*HLA-A*	3105	T>A,C,G	2571381
		T>A,C,G	2499
*LZTFL1*	54585	A>G	35044562
	54716	del/A	11385942
*OAS3*	4940	G>A,C	10735079
*TP53*	7157	G>A,C,T	1042522

All the details are according to https://www.ncbi.nlm.nih.gov/snp/.

To investigate possible associations between genetic determinants and anti-SARS-CoV-2 vaccine-induced antibody levels, the following statistical evaluations have been performed in the whole cohort and separately for the two vaccine types (when not shown, data comparison did not reach statistical significant values or trends in any of the subgroups considered).

**FIGURE 3:**
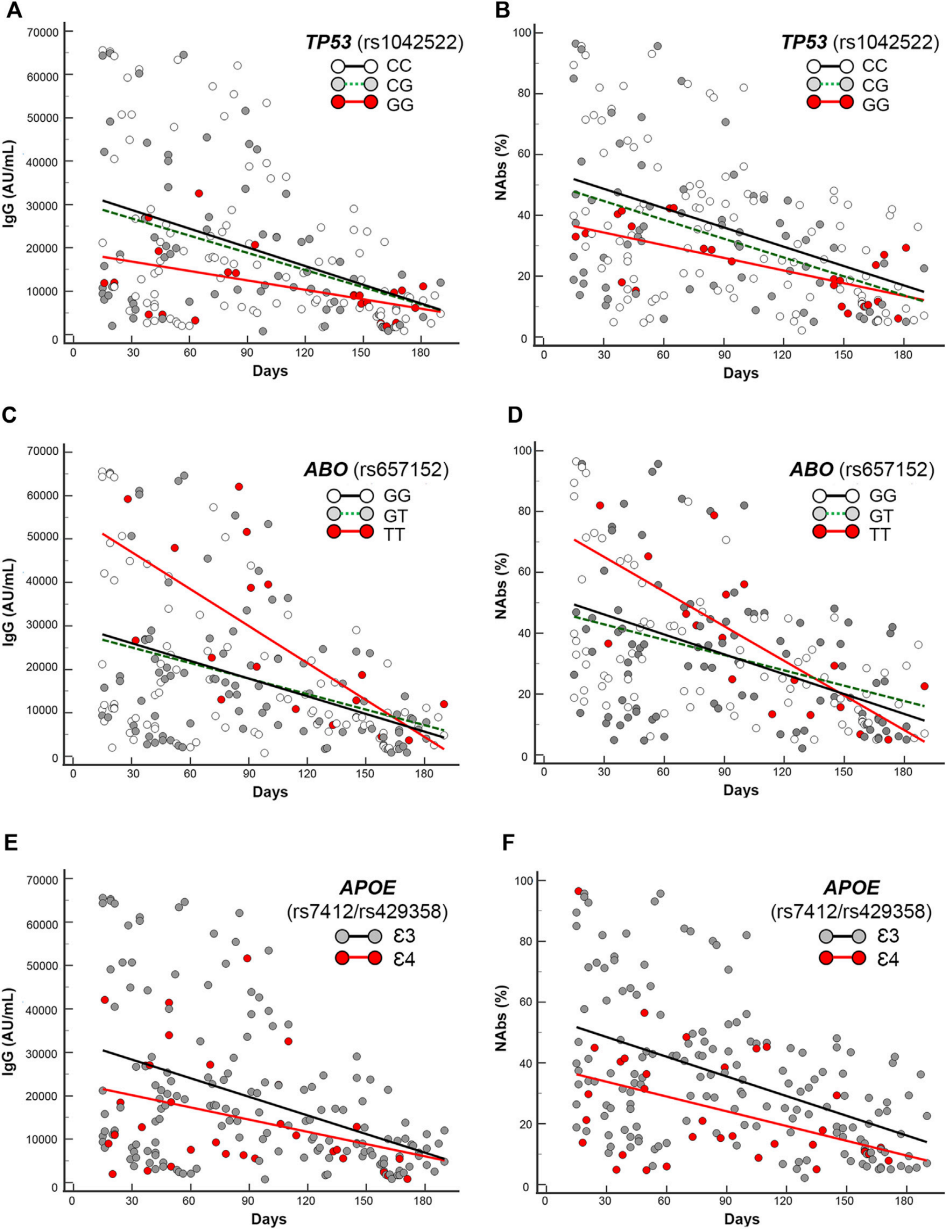
Scatter plots and regression analyses of IgG and NAbs levels stratified by the selected gene variants in the whole cohort. **(A,C,E)** IgG and **(B,D,F)** NAbs kinetics according to *TP53* (rs1042522), *ABO* (rs657152), and *APOE* (rs7412/rs429358). Each panel shows the specific regression lines, according to the indicated genotype/haplotype.

**FIGURE 4:**
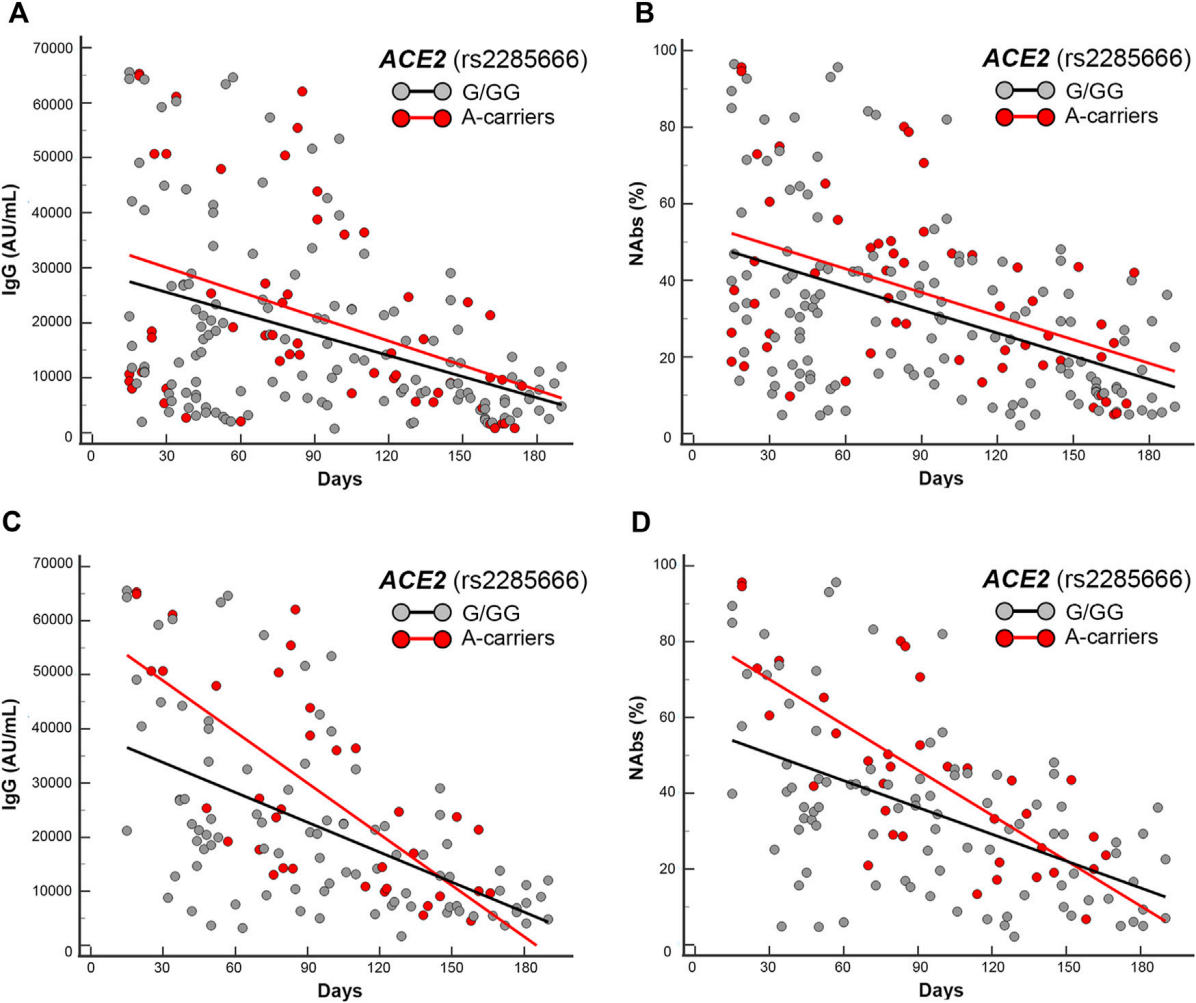
Scatter plots and regression analyses of IgG and NAbs levels stratified by *ACE2* (rs2285666) genotypes. **(A,C)** IgG and **(B,D)** NAbs kinetics in the whole cohort **(A,B)** and in the mRNA-based vaccine subgroup **(C,D)**. Each panel shows the specific regression lines, according to the indicated genotype.

**FIGURE 5:**
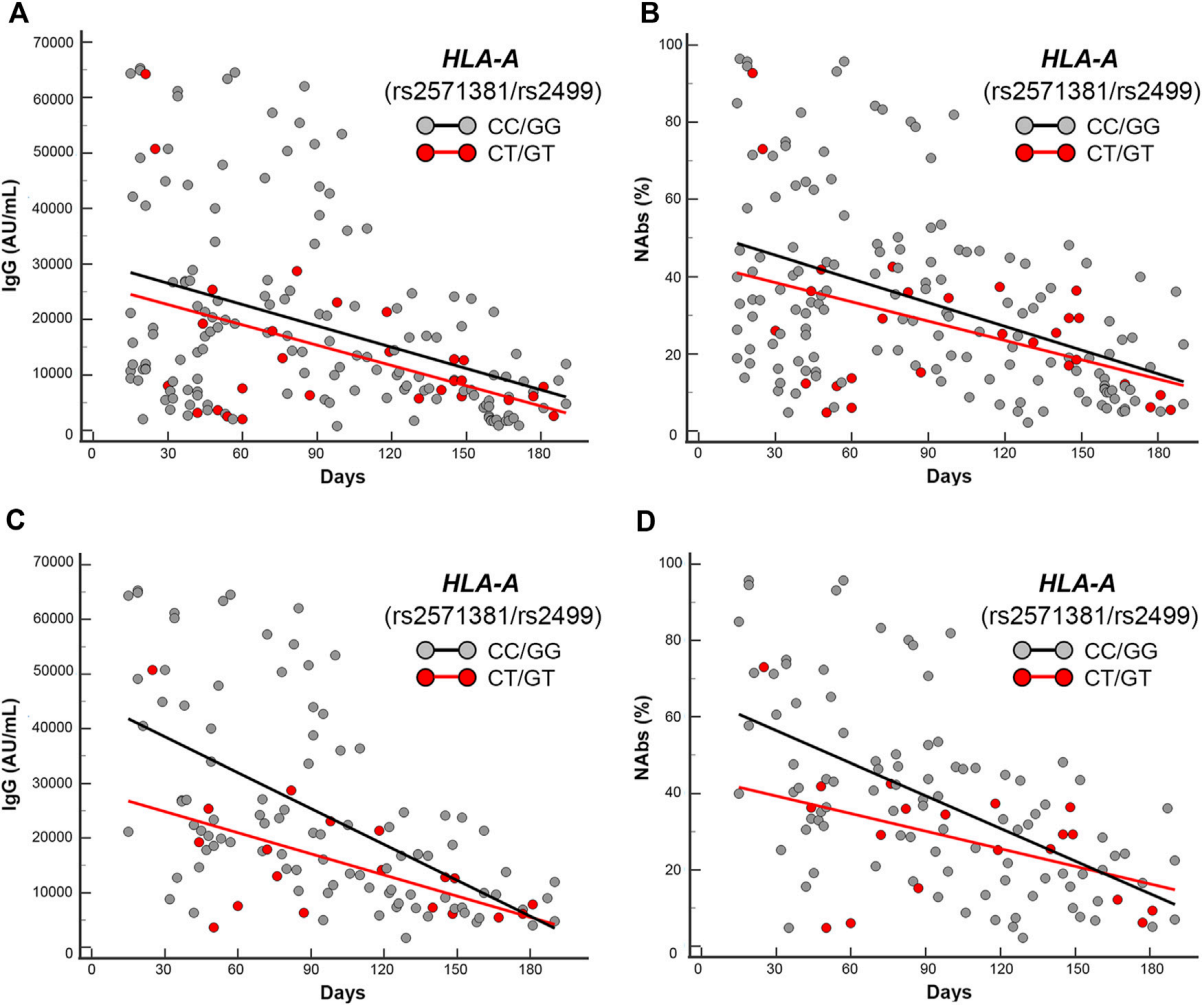
Scatter plots and regression analyses of IgG and NAbs levels stratified by *HLA-A* (rs2571381/rs2499). **(A,C)** IgG and **(B,D)** NAbs kinetics in the whole cohort **(A,B)** and in the mRNA-based vaccine subgroup **(C,D)**. Each panel shows the specific regression lines according to the indicated haplotype.

First, by regression analysis we performed the trend estimation of IgG and NAbs levels in the T1–T2 period of 190 days, accounting for the different subgroups of genotypes. The intercepts and slopes comparison of the regression equations of the different subgroups of genotypes yielded significant parameters computing the following gene variants or haplotypes: *TP53* (rs1042522), *ABO* (rs657152), *APOE* (rs7412/rs429358), *ACE2* (rs2285666), *HLA-A* (rs2571381/rs2499), and *CRP* rs2808635/rs876538. The related findings are shown in Figures 3A–F, Figures 4A–D, Figures 5A–D, and Supplementary Figures S2. We describe in detail the statistical parameters for regression analyses in the whole group as follows, and Table 3 (whole cohort) and Supplementary Table S1 (mRNA-subgroup) list the complete regression parameters of each comparison (IgG and NAbs).

**TABLE 3:** Regression analysis and genotype comparison of the selected SNPs in the whole cohort.

Gene (rs)		*TP53* rs1042522		*ABO* rs657152		*APOE* rs7412/rs429358		*ACE2* rs2285666		*HLA-A* rs2571381/rs2499	
Genotype/haplotype		CC + GC	GG	GG + GT	TT	Ɛ3	Ɛ4	G + GG	A-carriers	CC/GG	CT/GT
**IgG**	***r*** ^***2***^	0.1634	0.2545	0.1666	0.4562	0.1885	0.1175	0.1749	0.1694	0.1554	0.1987
	**Intercept**	32,036.5	19,014.1	29,424.6	55,512.2	32,608.5	22,957.4	29,393.1	34,509.9	30,400.7	26,360.5
	**Slope**	-138.4	-72.6	-127.7	-283.6	-142.8	-93.9	-127.8	-148.4	-128.34	-122.15
	***P*** intercept	<0.0001	<0.0001	<0.0001	<0.0001	<0.0001	<0.0001	<0.0001	<0.0001	<0.0001	<0.0001
	***P*** slope	<0.0001	0.0008	<0.0001	0.002	<0.0001	0.025	<0.0001	0.0016	<0.0001	0.017
	***P*** comparison*^a^*	**0.03**		**0.01**		**0.03**		0.09		0.2	
**NAbs**	***r*** ^***2***^	0.198	0.466	0.199	0.557	0.2432	0.1588	0.2157	0.2168	0.2090	0.1934
	**Intercept**	53.19	38.53	50.44	76.48	55.03	36.69	50.47	55.38	51.71	43.49
	**Slope**	-0.2071	-0.139	-0.1936	-0.38	-0.216	-0.16	-0.202	-0.206	-0.2049	-0.1668
	***P*** intercept	<0.0001	<0.0001	<0.0001	<0.0001	<0.0001	<0.0001	<0.0001	<0.0001	<0.0001	<0.0001
	***P*** slope	<0.0001	0.0002	<0.0001	0.0004	<0.0001	0.020	<0.0001	0.0003	<0.0001	0.019
	***P*** comparison*^a^*	**0.04**		**0.03**		**0.002**		0.075		0.2	

^a^ ***P*** *comparison* indicates the statistical assessment between the regressions obtained by the homozygous rare variant vs. the regressions of the homozygous common variant coupled with the heterozygous carriers (recessive model), in case these two latter significantly overlapped as shown in Figures 3A–D (i.e., *TP3* GG vs*.* CC + CG, and *ABO* TT vs*.* GG + GT). In the case of *APOE*, (Ɛ3-haplotypes vs*.* Ɛ4-carrying haplotypes) and in the case of *HLA-A*, (GG/CC, haplotype vs*.* CT/TG, haplotype) have been compared. *ACE2* comparison accounted for the absence of the A-allele (i.e., G-males + GG-females) vs*.* the presence of the A-allele (i.e., A-males + AA-females + AG-females). In bold significant *p*-values (comparison).

***TP53*** rs1042522 **GG**-genotype yielded significant regression equations clustered in the lowest part of the scattering when compared with the regression obtained by the rest of genotypes (CC + CG), accounting for significant different intercepts comparisons (*p* = 0.03 and *p* = 0.04, respectively, for IgG and NAbs distributions in the whole cohort, and *p* = 0.035 and *p* = 0.05, respectively, for IgG and NAbs distributions in the mRNA vaccine subgroup).

***ABO*** rs657152 **TT**-genotype yielded significant regression equations clustered in the highest part of the scattering when compared with the regression obtained by the rest of genotypes (GG + GT), accounting for different intercepts comparisons (*p* = 0.01 and *p* = 0.03, respectively, for IgG and NAbs distributions in the whole cohort, and *p* = 0.03 and *p* = 0.04, respectively, for IgG and NAbs distributions in the mRNA vaccine subgroup).

***APOE*** rs7412/rs429358 **Ɛ4**-carrying haplotypes (i.e., Ɛ2Ɛ4 and Ɛ3Ɛ4) yielded significant regression equations clustered in the lowest part of the scattering when compared with the regression obtained by the rest of **Ɛ3**-carrying haplotypes (i.e., Ɛ2Ɛ3 + Ɛ3Ɛ3), accounting for different intercept comparisons (*p* = 0.03 and *p* = 0.002, respectively, for IgG and NAbs distributions in the whole cohort, and *p* = 0.0018 and *p* = 0.0002, respectively, for IgG and NAbs distributions in the mRNA vaccine subgroup).

***ACE2*** rs2285666 **GA**-variant locates on the X chromosome, for this reason it was first analyzed in females and males separately, and both regressions yielded lower trends in the presence of the G-allele (i.e., GG-homozygous females or G-hemizygous males) when compared with the regressions obtained by the respective rest of genotypes (i.e., AA-homozygous in addition to AG-heterozygous females or A-hemizygous males). Accordingly, the combined sex analysis accounted for different intercept comparisons (*p* = 0.09 and *p* = 0.075, respectively, for IgG and NAbs distributions in the whole cohort) becoming significant (*p* = 0.014 and *p* = 0.009, respectively, for IgG and NAbs distributions) in the mRNA vaccine subgroup. In the sex-specific sub-analysis, only males maintained a significant intercept comparison within the mRNA vaccine subgroup (*p* = 0.01 and *p* = 0.0012, respectively, for IgG and NAbs distributions).

***HLA-A* CT/GT** variants (rs2571381/rs2499) are in strong/complete linkage disequilibrium, according to the different population analyzed (e.g., *r* ^2^ = 0.225244, *D*’ = 0.999985 and *r* ^2^ = 0.922353, *D*’ = 1.000000 from 1000GENOMES:phase_3 LWK and CEU for African ancestry and European ancestry origin, respectively). Accordingly, the haplotypes selected by the researchers to be compared have been the common double homozygotes (i.e., CC/GG) *versus* those carrying at least two variant alleles (e.g., CT/GT, TT/GG, CC/TT, CT/TT, and TT/GT). Due to the disequilibrium and the low frequency of double carriers, we found only double heterozygotes (i.e., CT/GT) and very few CT/TT. Regressions analysis yielded significant trends among double carriers (CT/GT + CT/TT) that clustered in the lower part of the scattering when compared with the regression obtained by the double homozygotes (CC/GG), accounting for significant different intercept comparisons only in the mRNA-based vaccine subgroup (*p* = 0.02 and *p* = 0.03, respectively, for IgG and NAbs distributions).

***CRP* TG/CT** variants (rs2808635/rs876538) are in strong linkage disequilibrium (e.g., *r* ^2^ = 0.618598, *D*’ = 0.965325 from 1000GENOMES:phase_3 CEU for European ancestry). Accordingly, the haplotypes selected by the researchers to be compared have been the common double homozygotes (i.e., TT/CC) *versus* those being at least polymorphic homozygous in any of the two loci (i.e., GG rs2808635 plus any combination of rs876538 or TT rs876538 plus any combination of rs2808635). Regressions analysis yielded significant trends among the polymorphic homozygotes (GG rs2808635/any rs876538 or TT rs876538/any rs2808635) that clustered in the higher part of scattering (Supplementary Figures S2A,B) when compared with regression obtained by the common double homozygotes (TT/CC), accounting for significant different intercept comparisons only in the mRNA-based subgroup for the IgG distribution (*p* = 0.01 and *p* = 0.09, respectively, for IgG and NAbs distributions).

Second, as a result of the significant difference in antibody levels between the two vaccine types and of the expected time-dependent fall in levels, we specifically analyzed the mean levels of IgG and NAbs in the T1 and T2 time points in the two cohorts of vaccines stratified by the different genotypes of the specific polymorphisms investigated. The antibody levels stratified by the genotypes gave appreciable and consistent differences in both subgroups of vaccine formulations, and among the AdV-based vaccine, the antibody mean gaps were even higher and retained the same trends observed among the mRNA-based vaccine though the lower number of cases in the former did not allow reaching the statistical significance. For this reason, we report the main findings among the mRNA vaccine subgroup as follows, and Table 4 shows in detail the mean *p*-values of the whole comparisons.

**TABLE 4:** IgG and NAbs levels stratified by vaccine formulation and by genotypes.

Vaccine	Gene	Genotype(s)								*p* values		
	***TP53***	**CC**		**CG**		**GG**		**CC+CG**		***p***_***1***_	***p***_***2***_	***p***_***3***_
		Mean ± SD	(n)	Mean ± SD	(n)	Mean ± SD	(n)	Mean ± SD	(n)			
**mRNA based**	IgG (T1)	33445.9 ± 20469.0	(33)	34205.5 ± 18659.6	(22)	19542.6 ± 10019.9	(7)	33.749.7 ± 19591.6	(55)	**0.04**	**0.03**	**0.03**
	NAbs (T1)	50.56 ± 26.32		48.14 ± 24.56		37.2 ± 6.10		49.6 ± 25.43		0.09	0.12	0.10
	IgG (T2)	16135.8 ± 12326.0	(37)	17048.7 ± 11.115.4	(21)	10149.2 ± 4568.9	(8)	16466.3 ± 10164.6	(58)	0.09	**0.05**	0.07
	NAbs (T2)	28.24 ± 18.23		30.21 ± 16.40		18.45 ± 9.25		28.96 ± 15.56		0.07	**0.03**	**0.05**
**Vector based**	IgG (T1)	13110.3 ± 14109.9	(21)	13356.7 ± 13117.0	(15)	8269.9 ± 4228.8	(4)	13212.9 ± 13513.1	(36)	0.25	0.23	0.23
	NAbs (T1)	34.78 ± 24.11		32.46 ± 26.20		25.05 ± 9.79		33.81 ± 24.66		0.22	0.29	0.24
	IgG (T2)	4857.9 ± 2829.4	(11)	3018.5 ± 2452.5	(10)	4491.1 ± 3489.1	(6)	3981.9 ± 2865.1	(21)	0.41	0.16	0.35
	NAbs (T2)	18.13 ± 12.69		11.13 ± 8.42		13.09 ± 3.66		14.80 ± 6.92		0.18	0.30	0.35

	***ABO***	**GG**		**GT**		**TT**		**GG + GT**		***p***_***1***_	***p***_***2***_	***p***_***3***_
		Mean ± SD	(n)	Mean ± SD	(n)	Mean ± SD	(n)	Mean ± SD	(n)			
**mRNA based**	IgG (T1)	33099.8 ± 18949.4	(26)	29217.9 ± 19115.7	(30)	42766.0 ± 20167.0	(6)	31020.2 ± 18965.9	(56)	0.13	0.06	0.06
	NAbs (T1)	49.9 ± 23.22		44.55 ± 26.02		59.0 ± 19.06		47.05 ± 24.69		0.19	0.1	0.13
	IgG (T2)	14463.6 ± 9466.39	(27)	16444.8 ± 12638.99	(29)	16881.8 ± 12928.39	(10)	15489.6 ± 12543.13	(56)	0.26	0.46	0.36
	NAbs (T2)	26.60 ± 15.25		29.98 ± 18.47		23.92 ± 17.79		28.35 ± 18.26		0.32	0.18	0.22
**Vector based**	IgG (T1)	13003.7 ± 15190.0	(19)	11749.0 ± 10670.7	(20)	26694.8	(1)	12360.2 ± 12909.0	(39)	-	-	-
	NAbs (T1)	35.24 ± 25.51		30.56 ± 22.83		36.63		32.84 ± 23.97		-	-	-
	IgG (T2)	4337.7 ± 2955.2	(12)	3649.1 ± 2819.3	(14)	7426.9	(1)	3966.9 ± 2886.6	(26)	-	-	-
	NAbs (T2)	13.83 ± 7.58		14.20 ± 11.92		24.58		14.03 ± 11.79		-	-	-

	***ACE2***	**A + AA**		**GA**		**G + GG**		-		***p***_***1***_	***p***_***2***_	-
		Mean ± SD	(n)	Mean ± SD	(n)	Mean ± SD	(n)	-	**-**			
**mRNA based**	IgG (T1)	42068.5 ± 28382.8	(4)	37513.2 ± 17940.6	(14)	23804.6 ± 18675.1	(44)	-	-	0.14	0.08	-
	NAbs (T1)	75.2 ± 27.07		49.5 ± 19.98		40.6 ± 24.40		-	-	**0.01**	0.1	-
	IgG (T2)	30354.3 ± 17075.8	(4)	10689.3 ± 10472.1	(14)	11715.2 ± 10801.2	(48)	-	-	**0.01**	0.3	-
	NAbs (T2)	45.20 ± 26.44		24.56 ± 12.13		25.42 ± 17.16		-	-	**0.04**	0.3	-
**Vector based**	IgG (T1)	8032.8 ± 5981.6	(6)	13638.1 ± 5015.8	(6)	8148.8 ± 15017.1	(28)	-	-	0.2	**0.045**	-
	NAbs (T1)	22.44 ± 13.84		30.10 ± 12.64		25.71 ± 26.97		-	-	0.2	0.40	-
	IgG (T2)	1658.4 ± 2200.7	(4)	4460.4 ± 3853.8	(4)	2706.8 ± 2811.1	(19)	-	-	0.11	0.44	-
	NAbs (T2)	8.93 ± 7.98		13.73 ± 16.67		10.9 ± 8.98		-	-	0.29	0.21	-

	***APOE***	**Ɛ3**		**Ɛ4**		-		-		***p***	-	-
		Mean ± SD	(n)	Mean ± SD	(n)	-	-	-	**-**			
**mRNA based**	IgG (T1)	34546.8 ± 19466.4	(50)	22199.4 ± 15140.01	(12)	-	-	-	-	**0.02**	-	-
	NAbs (T1)	53.0 ± 23.28		28.3 ± 18.17		-	-	-	-	**0.0005**	-	-
	IgG (T2)	15959.0 ± 11646.0	(58)	13826.9 ± 9376.8	(8)	-	-	-	-	0.3	-	-
	NAbs (T2)	28.32 ± 17.28		23.06 ± 14.86		-	-	-	-	0.21	-	-
**Vector based**	IgG (T1)	12632.3 ± 12949.4	(33)	13125.7 ± 3936.1	(7)	-	-	-	-	0.4	-	-
	NAbs (T1)	32.8 ± 22.69		33.8 ± 29.92		-	-	-	-	0.4	-	-
	IgG (T2)	4210.0 ± 2922.17	(22)	3589.3 ± 2877.02	(5)	-	-	-	-	0.3	-	-
	NAbs (T2)	15.44 ± 10.68		9.9 ± 4.05		-	-	-	-	0.1	-	-

	***HLA***	**CC + GG**		**CT + GT**		-		-		***p***	-	-
		Mean ± SD	(n)	Mean ± SD	(n)	-	-	-	**-**			
**mRNA based**	IgG (T1)	34249.0 ± 18.559.2	(47)	19186.7 ± 14.595.4	(10)	-	-	-	-	**0.01**	-	-
	NAbs (T1)	50.6 ± 23.63		31.6 ± 21.32		-	-	-	-	**0.015**	-	-
	IgG (T2)	15730.2 ± 11.709.5	(49)	11691.5 ± 6.372.8	(10)	-	-	-	-	0.14	-	-
	NAbs (T2)	27.13 ± 17.82		24.51 ± 11.44		-	-	-	-	0.33	-	-
**Vector based**	IgG (T1)	12084.9 ± 10.581.9	(32)	16000.3 ± 27.086.9	(5)	-	-	-	-	0.27	-	-
	NAbs (T1)	32.17 ± 23.09		31.27 ± 34.84		-	-	-	-	0.46	-	-
	IgG (T2)	3520.0 ± 2.550.8	(21)	6554.0 ± 3.069.7	(4)	-	-	-	-	0.099	-	-
	NAbs (T2)	13.2 ± 8.86		15.9 ± 7.46		-	-	-	-	0.2	-	-

IgG and NAbs levels are assessed as AU/mL and AU% respectively; *p p*_*1*_ *p*_*2*_ *p*_*3*_ indicate the statistical mean comparisons among the different genotype subgroups. In bold significant *p*-values.

***Tp53*** rs1042522 **GG**-genotype yielded significant or borderline (IgG, *p* = 0.03 and NAbs, *p* = 0.09) lower mean antibody levels at T1 (IgG: 19542.6 AU/mL and NAbs: 37.2 AU%) when compared with the counterpart genotypes who instead showed overlapped antibody mean levels (IgG: *CC* 33445.9 AU/mL and *CG* 34205.5 AU/mL and NAbs: *CC* 50.56 AU% and *CG* 48.14 AU%). The mean levels comparison at T2 maintained similar trends characterized by borderline significant values.

***ABO*** rs657152 **TT**-genotype yielded appreciable or borderline (IgG, *p* = 0.06 and NAbs, *p* = 0.1) higher mean antibody levels at T1 (IgG: 42766.0 AU/mL and NAbs: 59.0 AU%) when compared with the counterpart genotypes (IgG: *GG* 33099.8 AU/mL and *GT* 29217.9 AU/mL and NAbs: *GG* 49.9 AU% and *GT* 44.55 AU%). The mean levels comparison at T2 was far from being statistically significant.

***APOE*** rs7412/rs429358 **Ɛ4**-carrying haplotypes (i.e., Ɛ2Ɛ4 or Ɛ3Ɛ4) yielded significant (IgG, *p* = 0.02 and NAbs, *p* = 0.0005) lower mean antibody levels at T1 (IgG: 22199.4 AU/mL and NAbs: 28.3 AU%) when compared with the counterpart haplotypes (i.e., Ɛ2Ɛ3 + Ɛ3Ɛ3), overall including those who did not carry the ε4-allele (IgG: 34546.8 AU/mL and NAbs: 53.0 AU%). The mean levels comparison at T2 maintained similar trends far from being statistically significant. 

***ACE2*** rs2285666 **GA**-variant, for the aforementioned reason (i.e., X-chromosome location), was first analyzed in females and males separately, and in both sexes the presence of the G-allele (i.e., GG-homozygous females or G-hemizygous males) gave lower mean antibody levels in T1 when compared with the opposite respective genotypes (i.e., AA-homozygous females or A-hemizygous males). In detail, the combined sex analysis yielded significant differences, though non-significant for IgG (*p* = 0.08) and significant for NAbs (*p* = 0.01), by genotypes comparison (IgG: *GG*-females + *G*-males 23804.6 AU/mL and NAbs: *GG*-females + *G*-males 40.6 AU%) being lower than (IgG: *AA*-females + *A*-males 42068.5 AU/ml and NAbs: *AA*-females + *A*-males 75.2 AU%). Accordingly, *AG*-heterozygous females showed intermediate antibody levels between those shown by the AA- and GG-homozygous females. Interestingly, the same comparisons maintained comparable trends and also significant *p*-values in T2.

***HLA-A*** CT/GT-haplotype (rs2571381/rs2499), for the aforementioned reason (i.e., strong linkage disequilibrium), was compared against the common double homozygote (i.e., CC/GG) yielding significant (IgG, *p* = 0.01; NAbs, *p* = 0.015) lower mean antibody levels at T1 (IgG: 19186.7 AU/mL and NAbs: 31.6 AU%) when compared with the counterpart CC/GG-haplotype (IgG: 34249.0 AU/mL and 50.6 AU%). The mean level comparisons in T2 maintained similar trends though characterized by not significant values.

***CRP* TG/CT** variants (rs2808635/rs876538), for the aforementioned reason (i.e., strong linkage disequilibrium) were compared selecting the common double homozygotes (i.e., TT/CC) vs. those being at least polymorphic homozygous in any of the two loci (i.e., GG rs2808635 plus any combination of rs876538 or TT rs876538 plus any combination of rs2808635). The common TT/CC haplotype yielded significant (IgG, *p* = 0.025; NAbs, *p* =0.045) lower mean antibody levels at T1 only in the mRNA vaccine subgroup (IgG: 27992.7 AU/mL and NAbs: 45.0 AU%) when compared with the counterpart GG/TT rare haplotype defined as earlier (IgG: 37339.6 and NAbs: 53.0 AU%); for these reasons, we report only these data here.

Finally, we computed how the genotype, haplotype, or allele frequency of the investigated gene variants stratified above and below the trend lines of the IgG or NAbs distribution in the whole cohort (Table 5) and in the mRNA vaccine subgroup (Supplementary Table S2) and also tested for possible deviation from the expected Hardy–Weinberg equilibrium.

**TABLE 5:** Genotype, allele or haplotype frequency of the selected gene variants in the area below and above the trend lines of the IgG and NAbs distribution in the whole cohort.

**IgG**	***TP53* rs1042522**			***ABO* rs657152**			***APOE* rs7412/rs429358**		***ACE2* rs2285666**		***HLA-A* rs2571381/rs2499**	
	***GG***	***GC***	***CC***	***TT***	***GT***	***GG***	ε ***4***	ε ***3***	***G***	***A***	***CC/GG***	***CT/GT***
*Low*	0.183	0.348	0.469	0.052	0.478	0.469	0.226	0.774	0.774	0.226	0.78	0.22
*High*	0.05	0.35	0.60	0.15	0.475	0.375	0.075	0.925	0.632	0.368	0.90	0.10
***P*** *-genotype*	**0.018**			**0.05**			—		—		—	
***P*** *-recessive*	**0.0064**			**0.02**			—		—		—	
***P*** *-dominant*	0.07			0.18			—		—		—	
***P*** *-allele*	**0.0054**			**0.045**			—		**0.03**		—	
***P*** *-haplotype*	—			—			**0.005**		—		**0.04**	

**NAbs**	***TP53* rs1042522**			***ABO* rs657152**			***APOE* rs7412/rs429358**		***ACE2* rs2285666**		***HLA-A* rs2571381/rs2499**	
	***GG***	***GC***	***CC***	***TT***	***GT***	***GG***	ε ***4***	ε ***3***	***G***	***A***	***CC/GG***	***CT/GT***
*Low*	0.190	0.343	0.467	0.057	0.562	0.381	0.238	0.762	0.791	0.209	0.79	0.21
*High*	0.056	0.322	0.622	0.133	0.387	0.48	0.078	0.922	0.634	0.366	0.91	0.09
***P*** *-genotype*	**0.009**			**0.02**			—		—		—	
***P*** *-recessive*	**0.004**			0.06			—		—		—	
***P*** *-dominant*	**0.03**			0.12			—		—		—	
***P*** *-allele*	**0.0017**			0.73			—		**0.016**		—	
***P*** *-haplotype*	—			—			**0.0025**		—		**0.03**	

*Low* and *High*, indicate the genotype distribution (frequency) below and above the IgG and NAbs, trend lines respectively. *P-genotype, P-recessive, P-dominant, P-allele, P-haplotype* indicate the statistical assessment according to the comparison model applied: genotype distribution, recessive, dominant, allelic, or haplotype respectively.

***TP53*** rs1042522 genotypes were differently distributed in the area above and below the trend lines of the antibodies level scattering of the whole group (*p* = 0.018 and *p* = 0.009 for IgG and NAbs, respectively). The homozygous GG-genotype clustered in the area below the trend lines, accordingly GG-genotypes (*p* = 0.0064 and *p* = 0.004 for IgG and NAbs, respectively) and G-allele (*p* = 0.0054 and *p* = 0.0017 for IgG and NAbs, respectively) were significantly overrepresented when compared with the rest of genotypes (i.e., CC + CG) and the counterpart C-allele, respectively.

***ABO*** rs657152 genotypes were differently distributed in the area above and below the trend lines of the antibodies level scattering of the whole group (*p* = 0.05 and *p* = 0.02 for IgG and NAbs, respectively). The homozygous TT-genotype clustered in the area above the trend lines, accordingly TT-genotypes (*p* = 0.02 and *p* = 0.06 for IgG and NAbs, respectively) were overrepresented when compared with the rest of genotypes (i.e., GG + GT).

***APOE*** rs7412/rs429358 haplotype distribution gave an overrepresentation of the **Ɛ4-allele** in the area below the trend lines of the antibodies level scattering of the whole group, accordingly Ɛ4-carrying haplotypes (i.e., Ɛ2Ɛ4 + Ɛ3Ɛ4) were significantly overrepresented (*p* = 0.005 and *p* = 0.0025 for IgG and NAbs, respectively) when compared with the counterpart haplotypes (i.e., Ɛ2Ɛ3 + Ɛ3Ɛ3) overall those who did not carry the Ɛ4-allele.

***ACE2*** rs2285666 GA-variant, as reported previously, was first analyzed in females and males separately, and in both the sexes, the presence of the G-allele (i.e., GG-homozygous females or G-hemizygous males) was overrepresented in the area below the trend lines of the antibodies level scattering (*p* = 0.03 and *p* = 0.016 for IgG and NAbs, respectively) when compared with the counterpart A-allele (i.e., AA-homozygous in addition to AG-heterozygous females or A-hemizygous males).

***HLA-A*** CT/GT-haplotype (rs2571381/rs2499), for the aforementioned reason (i.e., strong linkage disequilibrium), was compared against the common double homozygotes (i.e., CC/GG) clustering in the area below the trend lines of the antibodies level scattering of the whole group, accordingly CT/GT-haplotype was significantly overrepresented in the area below the trend lines when compared with the counterpart CC/GG-haplotype (*p* = 0.04 and *p* = 0.03 for IgG and NAbs, respectively).

***CRP*** TG/CT variants (rs2808635/rs876538), for the aforementioned reason (i.e., strong linkage disequilibrium), were assessed by comparing the common double homozygotes (i.e., TT/CC) vs. those being at least polymorphic homozygous in any of the two loci (as detailed previously). Nonetheless, the overrepresentation of the TT/CC haplotype in the area below the trend lines (i.e., IgG: 68.2% vs*.* 31.8%; *p* = 0.119 and NAbs: 58.2% vs*.* 41.8%; *p* = 0.39) as a consequence of the low number of cases in the rare haplotype, the comparison did not reach the statistical significance. For this reason, we report only these data here.

## Discussion

Dissecting the intricate features of complex phenotypes (Singh et al., 2010; Gemmati et al., 2012; Gemmati and Tisato, 2023), as COVID-19 and the vaccine-induced immune response in different individuals, is an intricate task, being the result of genome–phenome combinations, and a specific OMICs (COVIDomics) approach is the best way to face them (Gemmati et al., 2019; Gemmati et al., 2020; Gemmati and Tisato, 2020; Milani et al., 2022). During COVID-19 pandemic and anti-SARS-CoV-2 vaccination campaign, unpredictable extreme phenotypes (clinical and laboratory) have been observed, suggesting that inherited traits and predispositions at an individual or population level may account for such manifestations. Our study is within the frame of an ongoing project belonging to the COVID-19 Host Genetic Initiative (HGI) named “*Extreme genotype comparison and extreme clinical phenotype comparison in CoV-2 patients: direct candidate genes–pathways and GWAS*” (https://www.covid19hg.org/partners/?partner=rec0CufBJdOxdaest). COVID-19 pandemic continues to be a major public health threat, especially in countries with low vaccination rates, and novel genes responsible for different susceptibility and severity have been identified by the HGI consortium (COVID-19 Host Genetics Initiative, 2022). Accordingly, extreme phenotypes (i.e., individual vaccine-induced immune response) after anti-SARS-CoV-2 vaccine have been deeply investigated in this study. Anti-viral antibody response is a measure of reactivity against the viral peptides, and this has mixed genetic and environmental contributions according to the individual genome landscape (Jonsson et al., 2017; Chung et al., 2019; Castro Dopico and Karlsson Hedestam, 2022). Genetic loci with a role in determining the extent of the humoral response to SARS-CoV-2 infection or vaccination or associated to post-vaccine side effects have not been completely recognized.

The main result of this study is that the extent of anti-SARS-CoV-2 IgG and NAbs response broadly varied among individuals and vaccine types, and their levels decreased significantly starting from 3 months after the second dose of vaccine (i.e., T2). The most common characteristics investigated, such as age, sex, ABO blood group, did not completely account for the interindividual variability. Accordingly, male sex had slightly lower antibody levels (IgG and NAbs) compared to females in the first trimester after the second vaccine dose (i.e., T1), and age indistinctly affected antibody levels in males and females though male sex reached lower values at T2, particularly because they are already presented with lower mean antibody levels at T1. Finally, ABO blood group gave inconsistent results, ascribing to the O group and A group overlapping regression trends and mean levels in-between the highest B group and the lowest AB group in both vaccine types as recently described in healthy donor populations (Nunhofer et al., 2022).

The observation that during the first 90 days after the second dose (T1) the antibody levels did not change significantly among the cases of our cohort (*r* ^*2*^ = 0.001 and *r* ^*2*^ = 0.012, respectively, for IgG and NAbs), made us confident in having performed the core comparisons and conclusions, especially in the T1 frame considering T2 distinctive of the antibody drop (*r* ^*2*^ = 0.305 and *r* ^*2*^ = 0.242, respectively, for IgG and NAbs).

The strong correlation found between IgG and NAbs levels in both the vaccines during the 6 months of time considered has been useful to analyze data considering the whole cohort and separately by the vaccine type. The need of disaggregated data analyses is of significant importance to understand the extent to which any considered variable is influencing the outcome, genetic characteristics and sex included. In addition, the apparently arbitrary setting of the 6 months’ frame (i.e., T1 and T2) to evaluate the antibody levels could be considered unfaithful to identify individual genetic predispositions of the vaccine immune response; this is because genotypes could randomly cluster in a particular percentile in that specific time regardless its own role of “genetic predictor” if any. For this reason, about 50% of those subjects who belonged to the T1 window randomly underwent a second blood drawing so that their T1–T2 mean time matched that of the whole cohort, and data obtained did not show significant differences when compared with that of the whole cohort.

To ascribe to specific genes and genotypes potential effects in predicting IgG and NAbs titer or duration, we focused on common gene variants previously investigated as modifiers of the clinical phenotype of COVID-19 or as possible influencers of antibody response after SARS-CoV-2 infection or vaccine.

First, we found that at longer time frames (i.e., T2), genetics lost its role in determining the antibody level after vaccination and regardless the gene variant considered antibody put down definitely around 150 days after vaccination, but this does not mean that after this time the protection from SARS-CoV-2 is lost.

Conversely, significant differences emerged in the first months of observation (i.e., T1) in six genes carrying the following gene variants [i.e., *TP53* (rs1042522), *ABO* (rs657152), *APOE* (rs7412/rs429358), *ACE2* (rs2285666), *HLA-A* (rs2571381/rs2499), and *CRP* (rs2808635/rs876538)]. The three complementary approaches we applied to assess possible genetically driven differences in antibody levels after vaccination gave consistent results ascribing to a definite genotype or haplotype different trend estimations (i.e., regression analysis), mean levels stratified by genotypes (i.e., Welch’s *t*-test), and genotype or allele frequencies stratified by antibody distributions including possible deviation from the Hardy–Weinberg equilibrium. Although a multiple test comparison should be considered and a larger cohort of subjects recruited, due to the high number of gene variants considered in a trait that appears to be polygenic, we went for a single-analysis approach considering the explorative purpose of the investigation. The selected approach may represent a limitation of the study deserving further investigations in larger cohorts.

*TP53* rs1042522 is the most common *TP53* SNP occurring at codon P72R (CCC>CGC) (Matlashewski et al., 1987). Large part of the investigations addressed its role on cancer (De Souza et al., 2021), but interesting correlations on metabolic pathways, immune cells, and immune response have been recently suggested in COVID-19 (Milani et al., 2022). Our results are in line with the differentially expressed role of P72- and R72-allele on immune checkpoint inhibitors and on the association of the G-allele or GG-genotype with imbalanced immune regulation (Dolgikh et al., 2022) and immune dysfunction, ascribing to *TP53* gene a crucial role in the immune response. In general, R72 carrying population is more susceptible to viral infection because the pro-inflammatory immune response is not as strong as the P72 population (Lodhi et al., 2021). Indeed, the P72 allele is overrepresented among the equatorial populations, where the immune challenge is greater. The innate immune response significantly differs in the P72 and R72 alleles, and this difference is regulated by a subset of genes known to control the inflammatory response, including some of the NFκB target genes, which are better activated by the P72 variant (Lodhi et al., 2021).

*ABO* (rs657152) was first described as a genetic susceptibility locus in patients with COVID-19 with respiratory failure suggesting a potential involvement also for the ABO blood group system, showing a higher risk in blood group A and a protective effect in blood group O as compared with other groups (Severe Covid et al., 2020; Venkataraman et al., 2022). Contextually, rs657152 was hypothesized as a determinant in COVID-19 prognosis and severity together with the genes of the RAS-pathway (Gemmati et al., 2020; Gemmati and Tisato, 2020). After vaccination programs, ABO blood group was investigated as potentially involved in the quality of the specific immune response against SARS-CoV-2 infection or vaccination, though no definite results have been obtained as discussed previously (Gil-Manso et al., 2022; Nunhofer et al., 2022; Sgherza et al., 2022). The association we found with the rs657152 variant strongly recalls the previous described connotations, though further investigations matched with the phenotypic ABO blood group are needed.

*APOE Ɛ4* haplotype (rs7412/rs429358) has been shown to associate with increased susceptibility to SARS-CoV-2 infection and COVID-19 mortality in previous genetic studies (Kuo et al., 2020; Kurki et al., 2021). *APOE Ɛ4* allele has been particularly investigated in neurodegenerative and cognitive impairment diseases, and indeed, severe COVID-19 and Alzheimer’s disease share common genetic and metabolic pathways via the *OAS1* gene and many risk factors strongly overlap (Tisato et al., 2018; Gemmati et al., 2020; Magusali et al., 2021). Intriguingly, several circulating serum proteins (e.g., ApoE) may adsorb specific SARS-CoV-2 peptides and alter their infection ability by mediating the ACE2 receptor. On the other hand, SARS-CoV-2 RBD can cause significant structural changes in ApoE lipoprotein and by hijacking the metabolic lipoprotein pathway it may facilitate cell entry (Yin et al., 2021). Contextually, the binding drastically changes the RBD epitope and such alterations can theoretically substitute ACE2 for ApoE in SARS-COV-2 interaction. Such interaction is further demonstrated by the decreased levels of LDL (ApoE included) during SARS-CoV-2 infection, ascribing to APOE Ɛ2-allele protective effects (Espinosa-Salinas et al., 2022). As there exist different affinities of the different APOE isoforms for the LDL receptors, similar changes and modifications might alike be responsible for different RBD-APOE interactions, affecting in turn the global immune response.

*ACE2* (rs2285666) is one of the most relevant SNPs in the gene influencing activity and levels of the receptor, it is a transition G8790A with the GG-genotype characterized by about 50% expression reduction compared to AA-genotype. ACE2 is the SARS-CoV-2 receptor cell entry by interacting with the RBD SARS-CoV-2 spike proteins. Differently expressed ACE2 levels and/or qualitative variants affecting the spike-receptor interaction have been investigated as partially responsible for the wide range of COVID-19 symptoms. *ACE2* gene is located on the X chromosome, accordingly males and females are necessarily characterized by different genotype architectures ascribing to male only the G- or A-hemizygous condition compared to female having the chance to carry the AG-heterozygosity. This suggested possible benefits for females against the risk of infection considering them more protected than males considered the at-risk sex (Gemmati et al., 2020). After vaccination, the neo-synthetized spike proteins migrate to the cell surface and protrude with a comparable native-like conformation to be recognized by the immune system and start the immune response. On the other hand, the vaccine-induced spike proteins can react with ACE2 receptors of the several neighboring cells, including platelets, and after cells death several free-floating spike proteins circulate in the blood and systematically interact with ACE2 receptors expressed by a variety of cells promoting complex internalization and degradation (Angeli et al., 2021; Watanabe et al., 2021). This proposed mechanism, together with the genetically driven ACE2 receptor expression (i.e., rs2285666), follows the same affinity interactions of the virus spike proteins. This may potentially influence the individual immune response and/or the epitope availability being responsible for the wide range of antibody levels detected after vaccination (Angeli et al., 2021; Watanabe et al., 2021).

*HLA-A* (rs2571381/rs2499) are the leading common SNPs at HLA-A*03:01 locus and have been recently associated to reactogenicity after Pfizer–BioNtech vaccine with the strongest signal association (*p* = 1.16E-12 and *p* = 1.72E-16 respectively) after GWAS extreme phenotype comparisons (Bolze et al., 2022). Although it is not certain whether A*03:01 is the causative allele due to linkage disequilibrium, SNPs imputation quality, and different population ancestry, further specific functional investigations are mandatory. Identifying which epitope of the vaccine-derived spike protein exactly interacts with HLA-A*03:01 locus could attribute any causality to the vaccine-associated adverse reactions or to the antibody production extent (Bolze et al., 2022; Venkataraman et al., 2022). We found different regression trends and antibody levels by comparing the double carriers (CT/GT rare alleles) *vs.* the double homozygotes (CC/GG common alleles), suggesting possible associations not only to reactogenicity but also in predicting the immune response after SARS-CoV-2 vaccination or infection. Due to the low frequency of the rare genotype combinations, we could not investigate the effect in the homozygotes for the rare alleles, the best candidates to show even greater genotype-associated differences, suggesting larger population studies.

*CRP* (rs2808635/rs876538) variants are associated to basal and stimulated circulating levels of CRP (Brull et al., 2003), and several investigations ascribed to these SNPs prognostic pharmacogenomics information on treatment and drug response (Parmeggiani et al., 2008; Parmeggiani et al., 2011; Moran et al., 2018). These molecular markers may be more than indicators of inflammation, and preferably, they can play a key role also in the response to external infectious stimuli. The interesting association we found with *CRP* rs2808635/rs876538 variants are in agreement with recent reports on CRP and anti-SARS-CoV-2 IgG levels. Baseline anti-SARS-CoV-2 IgG levels have been associated with CRP levels in COVID-19 patients, suggesting persistence of an unresolved inflammation status (Gianfagna et al., 2022). Moreover, among vaccinated subjects monitored after the second dose, those subjects showing a rapid IgG growth and a better kinetic pattern also had the highest CRP levels, considered as the best performers to anti-SARS-CoV-2 vaccination. The positive association between high CRP levels and post-vaccination IgG response has also been confirmed in a detailed survey of healthcare workers after Pfizer/BioNTech vaccine (Salvagno et al., 2022a). The authors found a highly significant correlation between pre-booster serum CRP levels and post-booster anti-SARS-COV-2 spike trimeric-RBD IgG antibodies. Conversely, no significant association was found between serum pre-booster levels of CRP and anti-SARS-CoV-2 spike trimeric IgG, concluding that the extent of the humoral response after COVID-19 vaccination might be affected by baseline inflammation as assessed by individual CRP levels.

The genetic markers we investigated may predict in advance those subjects at higher risk to be considered lower responders and could be used as valuable indicators for targeted vaccine booster administration and prevention programs. Overall, these results suggest that different genetic backgrounds may influence the vaccine-induced immune response and suggest that host genetics has a key role. Genetic differences, age, sex, and environmental factors can contribute to alter or enforce the immune response as demonstrated for other vaccines (Hamming et al., 2004; Gemmati and Tisato, 2020; Liao et al., 2020; Tay et al., 2020; Castro Dopico and Karlsson Hedestam, 2022; Milani et al., 2022). Combined COVIDomics investigative approaches may identify key determinants of the vaccine-induced immune response, suggesting possible genetic predispositions to develop a more efficient vaccine response. The new insights gained by using polygenic predictive markers merged with demographic data and serological screenings, may increase the understanding of the individual humoral response, accounting for ethnic and geographical differences, in both COVID-19 and anti-SARS-CoV-2 vaccination.

## Conclusion

Genetic predispositions and haplotypes fostering favorable immune responses might be helpful to recognize lowly or non-responsive individuals to any vaccination. Future larger and multicenter studies could help in elucidating the underlying inherited causes that could arise from a previous history of similar infections together with individual and environmental factors. The identification of specific genetic signatures in loci useful to track in advance those subjects at risk of a weak antibody response after vaccination or infection has an unquestionable translational power.

Extremely useful future implications of our genetic analyses can also be directed to dissect the molecular mechanisms underlying the individual humoral and cell-mediated variability to the vaccine(s) by *in vitro* dedicated experiments in relevant cell models. As recently reported, whole blood cultures from subjects after a single dose of anti-SARS-CoV-2 vaccine or after two doses of an adjuvanted SARS-CoV-2 recombinant protein also produced different cytokine secretion profiles (Lineburg et al., 2021; De Rosa et al., 2022). This paves the way to design candidate genomic screening and personalized timing to optimize dose administration in any vaccination program. Moreover, advanced 3D cell models based on integrated circuits and systems as chip technology may help and serve as an additional *in vitro* surrogate to summarize the complex response of any cell/organ target for the virus recapitulating the native cellular environments under higher biological fidelity (Singh et al., 2022). This will allow a more efficient and personalized approach for vaccine selection, formulation, timing, and scheduling of the booster doses.

Host genetic investigation is extremely useful when designing, selecting for specific peptide vaccines, and checking for their efficacy and protection across populations. However, it is likely that other important genetic influences and heritable traits exist together with acquired and concomitant situations that are responsible for the wide extent of the observed immune response.

## Data Availability

The original contributions presented in the study are included in the article/Supplementary Material, further inquiries can be directed to the corresponding author.
