# Supplementary material for: Host genetics impact on SARS-CoV-2 vaccine-induced immunoglobulin levels and dynamics: The role of TP53, ABO, APOE, ACE2, HLA-A, and CRP genes
Source: Front Genet. 2022 Nov 30;13:1028081. doi: 10.3389/fgene.2022.1028081 (PMC9748098; doi:10.3389/fgene.2022.1028081)
Supplement: Supplementary file 1 [file DataSheet1.docx]

Supplementary Material

# Supplementary Tables

| S**upplementary Table 1. Regression analysis and genotype comparison of the selected SNPs in the m-RNA based vaccine subgroup** | | | | | | | | | | | |
| --- | --- | --- | --- | --- | --- | --- | --- | --- | --- | --- | --- |
| **Gene (rs)** | | ***TP53***  rs1042522 | | ***ABO***  rs657152 | | ***APOE***  rs7412/rs429358 | | ***ACE2***  rs2285666 | | ***HLA-A***  rs2571381/rs2499 | |
| **Genotype/Haplotype** | | CC+GC | GG | GG+GT | TT | Ɛ3 | Ɛ4 | G+GG | A-carriers | CC/GG | CT/GT |
| **IgG** | **R^2^** | 0.3416 | 0.4677 | 0.3343 | 0.5362 | 0.3951 | 0.1177 | 0.3062 | 0.5102 | 0.3362 | 0.3066 |
|  | **Intercept** | 45757.2 | 26703.3 | 42410.0 | 62297.4 | 46902.0 | 28690.7 | 39334.9 | 58368.3 | 45111.1 | 28726.8 |
|  | **Slope** | -224.24 | -110.48 | -205.86 | -327.23 | -230.61 | -115.36 | -184.24 | -315.24 | -219.99 | -129.01 |
|  | ***P*-***intercept* | <0.0001 | <0.0001 | <0.0001 | <0.0001 | <0.0001 | <0.0001 | <0.0001 | <0.0001 | <0.0001 | <0.0001 |
|  | ***P*-***slope* | <0.0001 | 0.0049 | <0.0001 | 0.0013 | <0.0001 | 0.138 | <0.0001 | <0.0001 | <0.0001 | 0.01 |
|  | ***P-****comparison* | **0.035** | | **0.03** | | **0.0018** | | **0.014** | | **0.02** | |
| **NAbs** | **R^2^** | 0.3110 | 0.5951 | 0.2899 | 0.6575 | 0.4115 | 0.04807 | 0.2733 | 0.5511 | 0.3218 | 0.2006 |
|  | **Intercept** | 65.23 | 46.39 | 61.42 | 85.93 | 69.31 | 33.96 | 57.51 | 82.04 | 64.98 | 43.93 |
|  | **Slope** | -0.2817 | -0.1749 | -0.2550 | -0.4481 | -0.3054 | -0.09115 | -0.236 | -0.3989 | -0.28 | -0.1535 |
|  | ***P*-***intercept* | <0.0001 | <0.0001 | <0.0001 | <0.0001 | <0.0001 | 0.0014 | <0.0001 | <0.0001 | <0.0001 | 0.0001 |
|  | ***P*-***slope* | <0.0001 | 0.0008 | <0.0001 | 0.0001 | <0.0001 | 0.353 | <0.0001 | <0.0001 | <0.0001 | 0.05 |
|  | ***P-****comparison* | **0.05** | | **0.04** | | **0.0002** | | **0.009** | | **0.03** | |

***P*** *comparison*, indicated the statistical assessment between the regressions obtained by the homozygous rare variant *versus* the regressions of the homozygous common variant coupled with the heterozygous carriers (recessive model) in case these two latter significantly overlapped as shown in Figure 3 a-d (i.e. *TP3* GG *vs* CC+CG and *ABO* TT *vs* GG+GT). In the case of *APOE*, (Ɛ3-haplotypes *vs* Ɛ4-carrying haplotypes) and in the case of *HLA-A* (GG/CC haplotype *vs* CT/TG haplotype) have been compared. *ACE2* comparison accounted for the absence of the A-allele (i.e. G-males + GG-females) *vs* the presence of the A-allele (i.e. A-males + AA-females + AG-females). In bold significant P-values.

| **Supplementary Table 2. Genotype, allele or haplotype frequency of the selected gene variants in the area below and above the trend lines of the IgG and NAbs distribution in the m-RNA based vaccine subgroup** | | | | | | | | | | | | | |
| --- | --- | --- | --- | --- | --- | --- | --- | --- | --- | --- | --- | --- | --- |
| **Gene (rs)** | | ***TP53***  rs1042522 | | | ***ABO***  rs657152 | | | ***APOE***  rs7412/rs429358 | | ***ACE2***  rs2285666 | | ***HLA-A***  rs2571381/rs2499 | |
| **Genotype/Haplotype** | | GG | GC | CC | TT | GT | GG | Ɛ4 | Ɛ3 | G | A | CC/GG | CT/GT |
| **IgG** | *Low* | 0.182 | 0.303 | 0.515 | 0.076 | 0.454 | 0.47 | 0.227 | 0.773 | 0.789 | 0.211 | 0.773 | 0.227 |
|  | *High* | 0.048 | 0.371 | 0.581 | 0.177 | 0.468 | 0.355 | 0.080 | 0.920 | 0.627 | 0.373 | 0.918 | 0.082 |
|  | ***P****-genotype* | **0.05** | | | 0.08 | | | -- | | -- | | -- | |
|  | ***P****-recessive* | **0.018** | | | 0.08 | | | -- | | -- | | -- | |
|  | ***P****-dominant* | 0.4 | | | 0.8 | | | -- | | -- | | -- | |
|  | ***P****-allele* | 0.07 | | | 0.07 | | | -- | | **0.045** | | -- | |
|  | ***P****-haplotype* | -- | | | -- | | | **0.022** | | -- | | **0.037** | |
| **NAbs** | *Low* | 0.184 | 0.354 | 0.462 | 0.077 | 0.446 | 0.477 | 0.230 | 0.770 | 0.828 | 0.172 | 0.770 | 0.230 |
|  | *High* | 0.048 | 0.317 | 0.635 | 0.174 | 0.476 | 0.350 | 0.080 | 0.920 | 0.596 | 0.404 | 0.907 | 0.093 |
|  | ***P****-genotype* | **0.03** | | | 0.15 | | | -- | | -- | | -- | |
|  | ***P****-recessive* | **0.0159** | | | 0.09 | | | -- | | -- | | -- | |
|  | ***P****-dominant* | **0.048** | | | 0.14 | | | -- | | -- | | -- | |
|  | ***P****-allele* | **0.0059** | | | **0.05** | | | -- | | **0.01** | | -- | |
|  | ***P****-haplotype* | -- | | | -- | | | **0.0183** | | -- | | **0.04** | |

*Low* and *High*, indicate the genotype distribution (frequency) below and above the IgG and NAbs trend lines respectively. *P*-*genotype*, *P-recessive, P-dominant, P-allele, P-haplotype* indicate the statistical assessment according to the comparison model applied: genotype distribution, recessive, dominant, allelic, or haplotype respectively. In bold significant P-values.

## Supplementary Figures

**Supplementary Figure 1. Comparison between IgG and NAbs levels in the whole cohort and in the paired sampling subgroup.** (**A** and **B**) IgG and NAbs level distribution respectively on the m-RNA-based vaccine subgroup stratified by T1 and T2. (**C** and **D**) IgG and NAbs level distribution respectively on the Vector-based vaccine subgroup stratified by T1 and T2. Blue and red solid dots (T1) refer to the whole cohort and the paired samples respectively; blue and red empty dot (T2) refer to the whole cohort and the paired samples respectively. Horizontal lines represent median values and interquartile range for each group.

**Supplementary Figure 2. Scatter plots and regression analyses of IgG and NAbs levels stratified by *CRP* (rs2808635/rs876538).** (**A**) IgG and (**B**) NAbs kinetics in the m-RNA based vaccine subgroup. Each panel shows the specific regression lines according to the indicated haplotype.
